# The influencing factors of nurses' job engagement in tertiary, A grade hospitals in East China: A cross‐sectional study

**DOI:** 10.1002/nop2.70037

**Published:** 2024-09-23

**Authors:** Ling Li, Zhixian Feng, Mingling Zhu, Jialu Yang, Lili Yang

**Affiliations:** ^1^ School of Nursing Zhejiang Shuren University Hangzhou China; ^2^ School of Nursing Zhejiang Chinese Medical University Hangzhou China

**Keywords:** adversity quotient, job engagement, mental workload, nurses

## Abstract

**Aim:**

To explore the effects of demographic characteristics, mental workload, and Adversity quotient (AQ) on the job engagement of nurses in East China.

**Design:**

A quantitative and cross‐sectional study.

**Method:**

The survey collected questionnaire data on mental workload, adversity quotient, and job engagement from 473 nurses selected working in 12 Grade‐A tertiary hospitals based on informed consent in East China between July 2020 and March 2021.

**Results:**

The total score of mental workload was 78.24 ± 11.65, the adversity quotient score was 128.26 ± 15.84, job engagement score was 42.32 ± 7.79. Job engagement has a remarkable positive correlation with adversity quotient (*r* = 0.613, *p*<0.001), and a negative correlation with mental workload (*r* = −0.499, *p*<0.001). Mental workload has an apparent negative correlation with adversity quotient (*r* = −0.291, *p*<0.001). Labor‐management relationship with current organization, department, study to get a degree or diploma in spare time, attitude towards a career in Nursing, attitude towards the current career position, satisfaction with marriage, social support, load feelings, self‐assessment, control, and endurance could predict 70.9% of job engagement of nurses.

**Conclusions:**

The mental workload of nurses was higher, the AQ was at a medium level, and the job engagement of nurses was also slightly higher. Labor‐management relationship with current organization, department, study to get a degree or diploma in spare time, attitude towards a career in Nursing, attitude towards the current career position, satisfaction with marriage, social support, load feelings, self‐assessment, control, and endurance had predictive effects on nurses' job engagement. It is necessary to take a variety of measures according to the social‐demographic characteristics, improve the adversity quotient, and evaluate the mental workload correctly, to improve the job engagement of nurses.

**Impact:**

The epidemic situation and other emergencies make the work pressure of nurses in Grade‐A tertiary hospitals increase suddenly. It should pay attention to the influence of different demographic factors, and pay attention to the correct guidance of work demand‐mental workload, as well as the cultivation, and improvement of job resource‐AQ, which can improve the job engagement of nurses to some extent.

**Patient or Public Contribution:**

No patient or public contribution.

## 1 INTRODUCTION

The notice of document [2020] 7 of the National Health Commission on further strengthening the construction of the nurse team points out that the workload of nurses should be scientifically measured, the humanistic care of nurses should be strengthened, and the working environment, night shift conditions, and logistical support conditions should be improved, so that nurses can combine work and rest, and work comfortably and with peace of mind. It should also pay attention to the mental health of nurses, take various forms to strengthen psychological counselling, help nurses relieve pressure, and improve the quality of nursing services (National Health Commission., 2020).

In China, medical institutions can be divided into three types, community hospitals (level 1), secondary hospitals (Level 2) and tertiary hospitals (Level 3) according to grading standards (Jiang et al., 2023). Tertiary hospitals are the regional, or higher‐level hospitals that provide high‐level medical, and health services, and carry out higher education, and scientific research tasks in the region, and surrounding radiation area, which are more than 501 beds. A Grade is a grade with more than 900 points based on the scoring criteria. The requirements for staffing in a Grade‐A tertiary hospital are at least 1.03 health technicians per bed; With a minimum of 0.4 nurses per bed, it is the highest of the “three levels and six levels” of hospitals in mainland China, Different from community hospitals for specific communities, secondary hospitals provide comprehensive medical and health services for multiple communities; Tertiary hospitals provide high‐level, specialized medical and health services for the country people (Qin & Ding, 2021). Nurses in Grade‐A tertiary hospitals are usually equipped with solid nursing knowledge, and exquisite skills. Besides providing nursing services, they still have to undertake nursing teaching, scientific research, and other tasks. As a result, they have a heavier workload. The study found that the psychological stress and work burden of clinical nurses increased with the increase of hospital level (Wu et al., 2023). In addition, The incidence of PTSD in nurses was 1.92 times higher in tertiary hospitals than in secondary hospitals (Li et al., 2021).

During this pandemic, nurses in Grade‐A tertiary hospitals are facing greater work pressure than before in China. Nurses play a crucial part in preventing COVID‐19, preventing its spread, and providing nursing services for patients as one of the largest groups of healthcare providers in the country. But they face a higher risk of infection, and an increasing workload when providing care services to patients (Dai et al., 2020). A survey on nurses' job burnout after the epidemic found that emotional exhaustion, job apathy, and lack of job achievement were much more severe than their own before the outbreak in China, mainly considering that the nurses faced the double burden of routine treatment, epidemic prevention, and control during the fight against COVID‐19. Nurses must be on the front line to do many job contents, such as diagnosis, screening, medical observation, and specimen collection (Ke & Wang, 2020). In addition, some nursing staff had been sent out to support in other affected areas by the nursing administrators, which increased the work tasks, and psychological pressure of the left‐behind nurses indirectly, then increased job burnout. Nursing staff with a high sense of job burnout have low job effectiveness, and job engagement (Sun et al., 2023). However, regardless of the existence of the epidemic, nurses' job stress perception, and psychological resources affect their job engagement. The effect of different demographic characteristics, and mental workload (as a job requirement), AQ (as a job resource) on job engagement are not well understood.

### 1.1 Job engagement

Job engagement refers to the high degree of physical, and psychological involvement of individuals in activities, which means the degree to which a person identifies with their work psychologically, and the degree to which they attach importance to themselves (Zhao et al., 2021). Job engagement is a positive, fulfilling state of mind about work in the nursing profession, which is characterized by vigour, dedication, and absorption (Pan et al., 2022). Vigour means strong energy, mental resilience, and eagerness to commit exertion in one's worth of effort. Absorption refers to being wholly concentrated, joy in one's cost of effort, and having the difficulty of detaching oneself from work. Dedication is characterized as high levels of involvement in one's cost of effort, and a sense of meaningfulness, pride, challenge, inspiration, and enthusiasm (Peláez Zuberbühler et al., 2023). To solve the current situation of staff ratio, and workload imbalance, improve their job engagement, and promote their physical, and mental health, the job demands‐resources model (JD‐R model), which is an in‐depth study of job engagement, is utilized in this study. The JD‐R model is a model used by management to study work. It believes that work conditions are divided into job demands, such as physical, psychological, social, or organizational aspects of work requiring effort and, or skills. Job resources, such as physical, psychological, social, or administrative aspects of work help to achieve goals, stimulate personal growth, and reduce job demands. The employee distress levels, and engagement may be influenced by both of these job characteristics (Willems et al., 2021). The model emphasizes job demands, and job resources as predictors of burnout, and job engagement, respectively, which may affect the outcomes related to organization, and health in turn (Nordhall et al., 2020). The JD‐R model consists of two potential psychological processes: the health impairment process, and motivational processes. The health impairment process thinks that high job demands in the absence of job resources will cause distress. The motivational process predicts that the presence of job resources will contribute to higher engagement, and productivity (Sinval et al., 2022; Claes et al., 2023). Promoting job engagement is the most direct way to improve job performance effectively, and it can help to improve the quality of nursing, and ensure patient safety (Carthon et al., 2019; Parr et al., 2021; Zhang & Duan, 2023; Zhang & Peng, et al., 2023). “Buffer” hypothesis, holds that under high job resources, the negative impact of job requirements on employee job burnout is weakened, while under low job resources, the negative impact of job requirements on employee job burnout is enhanced (Cho et al., 2023). Previous research showed that success at work is associated with personal characteristics (e.g, job engagement, psychological capital, psychological contract) and relationship characteristics (e.g, perceived organizational support) (Gröschke et al., 2022; Huang et al., 2023; Kleine et al., 2019). Job engagement is affected by many factors, which can be divided into individual elements, and organizational factors (Kossyva et al., 2023). Personal factors include personal traits, occupational characteristics, family problems, job orientation, and personality factors include stress, self‐efficacy, and optimism (Merino‐Soto et al., 2022; Rattray et al., 2021; Wickström et al., 2020). Organizational factors mainly include realistic environment, and social support.

### 1.2 Mental workload

The mental workload of nurses refers to the psychological load that nurses bear when completing nursing tasks, including cognitive, and emotional loads. It is essential to guarantee the medical staff makes correct clinical decisions, and ensures patient safety when bearing appropriate psychological burdens (Deng et al., 2018). High workload, and stress affect job performance (Mattsson & Gustafsson, 2020). Job engagement is negatively correlated with workload (Ginbeto et al., 2023), especially with the frustration dimension. Nurses' perceived workload can predict their stress (Yen et al., 2019). A study found that workload is negatively correlated with job engagement. That is to say, a lack of human, and material resources will increase nurses' workload, thereby increasing job burnout, and reduce job engagement (Zhang, Chen, et al., 2023).

### 1.3 Adversity quotient

The adversity quotient (AQ) is an individual's emotional state, and reaction mode when encountering adversity. It indicates how individuals can manage difficulties, and turn obstacles into opportunities when facing failure, or frustration (Safi'i et al., 2021).AQ has four dimensions, control, ownership, reach, and endurance, namely, which constitute the CORE model. Among them, control refers to people's ability to control, and control the surrounding environment in the face of adversity, ownership refers to the cause of the occurrence of adversity, and the willingness to take responsibility, and improve the result. Reach refers to the extent, and the extent to which adversity affects other areas of life. Endurance refers to how long the hardship, and its causes affect a person's normal life (Liu et al., 2022). AQ can not only predict a person's career success, but also predict and influence all aspects of a person's ability, and performance (Stoltz 2000). It can be seen in a person's ability to maintain composure when facing problems (Safi'i et al., 2021). In particular, a high mental workload will increase physical fatigue, and mental stress in the clinical environment, hurting the job engagement of nurses potentially, the AQ may play a role in this. According to a review of previous studies, there was a significant positive correlation between AQ, and job engagement (Rahmayanti et al., 2020). AQ is an assessment of a person's ability to withstand adversity, and his/her ability to overcome any crisis (Wang, Ma, et al., 2022). According to stress appraisal theory (Lazarus & Folkman, 1984), the individual will form a perception of harm or loss, threat or challenge after primary appraisal of stress response. Secondary appraisal generates feelings or meanings attributed to specific events or situations, thereby enabling individuals to move from thinking to action (Regehr & Bober, 2005). Both injury or loss, and threat have negative attributes, but challenges are positive events. Challenge can also be defined as the potential for positive personal growth through the use of coping skills to reduce stressful events or encounters (Wang et al., 2020). Individuals perceive workload as a challenge, and choose to respond positively. Those who have a high AQ will put adversity in regard to a specific event, feel capable of handling it, and minimizing its negative influence (Kong et al., 2016), and perform more efficiently, and effectively (Safi'i et al., 2021). At the same time, the adversity quotient can also negatively predict suboptimal health (Xue et al., 2021). Therefore, it can be speculated that a high mental workload will increase the physical fatigue, and mental stress of nurses in the clinical environment, potentially damaging the job engagement of nurses. However, individuals with high AQ can withstand, buffer, and solve the workload, AQ may play a certain role in this. However, whether mental workload and AQ can play a role in nurses' job engagement and the extent need to be further discussed.

## 2 METHODS

### 2.1 Study purpose

The purpose of this study is to explore the effect of demographic characteristics, mental workload, and AQ on job engagement of Grade‐A tertiary hospital nurses in China, and provide a quantitative basis for improving their job engagement. This study examined three key research questions:

1. What is the current situation of nurses' mental workload, AQ, and job engagement?
2. What are the socio‐demographic factors that can influence nurses' job engagement?
3. what is the influence of mental workload (job requirements), and AQ (job resources) on nurses' job engagement and its extent?

### 2.2 Study design and participants

The study was designed as a cross‐sectional research adopting a convenient sampling method in East China. The participants were all registered nurses who were working in 12 hospitals in Hangzhou city of Zhejiang province. An explanation for the selection, and inclusion of study samples are provided in File S1. Inclusion criteria were as follows: (a) nurses who have obtained the registered nurse qualification certificate. (b) Nurses who were engaging in clinical nursing work. (c) Nurses who agreed to join in this study. Exclusion criteria included (a) nurses who were not primarily involved in patient care. (b) Nurses who could not participate in this study due to leaving or studying abroad during the survey period.

### 2.3 Data collection

The survey was conducted in Chinese, and online inquiry was achieved through the Questionnaire Star survey platform using self‐report questionnaires from July 2020 to March 2021. According to the calculation method of sample size in observational studies, the sample size should be 5 ~ 10 times the number of independent variables (Fang, 2012). This study included 21 socio‐demographic variables, which were designed by the research team, including 3 items of social demographic characteristics, eight items of work and study status, four items of professional and job attitude, five items of social and economic conditions, and one item of religious belief. Forty items of the AQ questionnaire, and six items of the mental workload questionnaire, sum up 67 independent variables, so the sample size was 335 cases. Considering the invalid questionnaire, the sample size was expanded by 10% (Wang, Yan, et al., 2022), and the required sample size was 369. The researchers sent the questionnaire links to 12 hospitals through acquaintances, and then sent it to their departments. After obtaining informed consent, respondents completed the questionnaire independently. Nurses who were not able to participate in this study may have been busy or not interested in participating. Questionnaire Star survey data need to be completed before submission, so no data is missing. After the data from the questionnaire platform was directly imported into SPSS, the researcher checked the filling of the data. It is determined by removing large differences between the mean response times, and when there are certain rules in the options, such as zigzag answers or the complete selection of a single number, which were considered valid questionnaires. A total of 25 discarded questionnaires were destroyed, and 473 questionnaires were obtained, with a valid questionnaire rate of 94.98%. The whole data analysis process is supervised by an associate professor who is proficient in statistics. The following are the instruments used in the study.

#### 2.3.1 NASA Mission load index scale for nurses

NASA Mission Load Index Scale for Nurses: The National Aeronautics and Space Administration Task Load Index (NASA‐TLX), developed by Hart (Hart & Staveland, 1988), is the subjective evaluation of mental load reported by many foreign studies, mostly used in the field of human ergonomics. The NASA task load index scale for nurses is formed through translation, back‐translation, and cultural adjustment by (Liang et al., 2019). It comprises six items: mental requirement, physical requirement, time limit requirement, effort level, self‐expression, and frustration. NASA‐TLX is divided into two dimensions: load feeling, and self‐assessment. Item score is marked with a line divided into 20 equal points representing 0–20 points, indicating mental workload from “low” to “high”; the item of “performance” is “perfect” to “failure” from left to right. The lower the score is, the more perfect the self‐performance, and the lower the workload is. The higher the score is, the more failed the self‐performance and the higher the workload is. Dimensions are divided into the sum of the corresponding item points, and the total score is the sum of all item points. The translated scale has good reliability and validity, and it's Cronbach's α coefficient is 0.707, and the retest reliability is 0.806. In this study, the Cronbach's α coefficient of the scale was 0.756.

#### 2.3.2 Job engagement scale

Job engagement scale: was developed by Schaufeli in 2006 and tested on health care workers, teachers and other populations in 10 countries. The total Cronbach α coefficient of the scale was 0.85 ~ 0.92 (Schaufeli et al., 2006). The nurses' job engagement scale translated by (Zhang & Gan) in 2005, with a Cronbachα coefficient of 0.930 and a Cronbachα coefficient of 0.740 ~ 0.900 for each dimension. The scale consists of nine items, and three dimensions, namely, vigour, absorption in one's work, and dedication, each of which contains three items. A Likert seven‐point scale was used for the scale, and 0–6 points were assigned to “never,” “hardly,” “rarely,” “sometimes,” “often,” “usually,” and “always.” The total score was 0–54. The higher the score means the higher the nurse's job engagement. The Cronbach's α coefficient of the scale was 0.854 in this study.

#### 2.3.3 The adversity quotient scale

The adversity quotient scale: This study adopts the adversity response profile, compiled by Paul Stoltz (1997), translated and revised by (Li & Chen, 2008), which mainly comprises four dimensions, control, ownership, reach, and endurance, including 20 types of adversity, and 40 entries. A 5‐point Likert scale was used for each item, with a total score of 40–200. The higher score the higher AQ. Stoltz divided the adversity quotient into five grades: very high (166–200), high (135–165), medium (95–134), low (60–94), and very low (< 59). The scoring standards of each dimension were as follows: low (10–23 points), medium (24–37 points), and high (38–50 points). According to the research of Dr. Li Bingquan and Chen Canrui, (Li & Chen, 2008) the overall Cronbach's α coefficient of the scale was 0.76, and the total score and retest reliability coefficients of the four parts were 0.72 ~ 0.79. The scale has been widely used in nursing students and nurses in China with good reliability and validity. In this study, it was 0.905.

### 2.4 Data analysis

The collected data were analysed using IBM SSPS 25.0. A descriptive statistical method was applied regarding the characteristics of the sample, and research variables. Categorical variables were expressed as numbers, and percentages, continuous variables were expressed as mean ± standard deviation (SD). Given the large sample size, according to the central limit theorem (Rivas er al, 2023), we assume that our sample follows the standard normal distribution. Statistical analysis was done using the Student's *t*‐test and one‐way analysis of variance (ANOVA) to compare sociodemographic characteristics, mental workload, and AQ on Multiple group comparisons of job engagement. The correlation analysis results among them were processed using Person correlation coefficients. The influencing factors of job engagement are discussed by using multiple linear regression analysis, p‐value <0.05 was regarded to be statistically significant.

### 2.5 Ethical considerations

The protocol for this study was approved by the Ethics Review Committee of the Institutional Review Board of a university. The research process was conducted under the Declaration of Helsinki, and its later amendments. Informed consent was obtained from participants before filling out the questionnaire, participants filled out the questionnaire anonymously, and the nurses were assured that they could withdraw at any time without penalty if they wished.

## 3 RESULTS

### 3.1 Participants and characteristics

The study sample is described in Table 1. Most respondents were female (*n* = 425, 89.85%). Most of them were 25 –35 years (51.59%). Those whose labor‐management relationship with the current organization having an establishment were the majority (*n* = 304, 64.27%), and length of service in nursing was mostly comprised of 2–5 years:114 (24.10%), 6–10 years: 120 (25.37%),11–20 years:112 (23.68%). A 30.23% proportion of respondents were in the internal medicine department. The majority (*n* = 415, 87.74%) had a Bachelor degree. Nurse‐in‐charge has the largest proportion (43.34%). Nurses with an annual salary of CNY100,000 to CNY150,000 yuan, and CNY160,000 to CNY200,000 yuan take up a larger proportion, accounting for 36.58%, and 35.10%, respectively. The proportion of married (*n* = 283, 59.83%) was larger than other marital statuses.62.37 percent of nurses were satisfied with their marriages. The proportion with good social support was 50.32%. The proportion of those with more financial pressure was slightly higher, at 30.87%. 79.28% of nurses had no significant stress in the last 6 months. The proportion of nurses with an average attitude towards a career in Nursing was slightly higher, at 46.30%. 45.67 percent of nurses were satisfied with their current career position. 58.77% of nurses think that it is tough for them to be promoted, and 80.55% of nurses think that nursing scientific research is complicated. The majority (39.53%) thought that opportunities for further study were average. 84.57 percent of nurses had no religion. The proportion of nurses who still study in their spare time to obtain a degree or diploma was relatively low, at 47.99 percent. Most of them are the non‐only children (62.37%). Table 1 presents the demographic characteristics of the participants.

**TABLE 1:** Background characteristics of the participants (*N* = 473).

Characteristics	*N*	%
**General demographic characteristics**		
Gender		
Male	48	10.15
Female	425	89.85
Age		
<25 years	84	17.76
25 years to 35 years	244	51.59
36 years and 40 years	89	18.82
>40 years	56	11.84
Are you an only child?		
Yes	178	37.63
No	295	
**Current status of work and study**		
Labor‐management relationship with the current organization		
Have an establishment	304	64.27
No establishment	169	35.73
Length of service in nursing		
<2 years	77	16.28
2–5 years	114	24.1
6–10 years	120	25.37
11–20 years	112	23.68
>20 years	50	10.57
Department of Work		
Internal medicine	143	30.23
Surgery	126	26.64
Outpatient clinic	19	4.02
Emergency	11	2.33
ICU	43	9.09
Paediatrics	17	3.59
Others	114	24.1
Highest level of education you currently have earned		
Technical secondary school degree	11	2.33
Associate degree	20	4.23
Bachelor degree	415	87.74
Master degree or above	27	5.71
Current Nursing Technical Title		
The nurse	75	15.86
Nurse practitioner	176	37.21
Nurse‐in‐charge	205	43.34
Associate chief nurse and above	17	3.59
Have you recently experienced significant stress? (within 6 months)		
Yes	98	20.72
No	375	79.28
Do you have opportunities for study and further education?		
A lot	32	6.77
More	156	32.98
Average	187	39.53
Less	82	17.34
None	16	3.38
Do you study to get a degree or diploma in your spare time		
Yes	227	47.99
No	246	52.01
**Attitude towards profession and position**		
Your Attitude towards a Career in Nursing		
Like it very much	34	7.19
Like it more	192	40.59
Average	219	46.3
Like it less	25	5.29
Dislike	3	0.63
Attitude towards the current career position		
Very satisfied	34	7.19
More satisfied	216	45.67
Average	195	41.23
Less satisfied	13	2.75
Dissatisfied	15	3.17
Do you think it's difficult to get promoted?		
Difficult	278	58.77
Average	183	38.69
Not difficult	12	2.54
Do you think Scientific research is stressful?		
Difficult	381	80.55
Average	83	17.55
Not difficult	9	1.9
**Social and economic situations**		
Current marital status		
Unmarried	164	34.67
Married	283	59.83
Divorced	9	1.9
Widowed	6	1.27
Remarried	11	2.33
Satisfaction with marriage		
Very satisfied	120	25.37
Be satisfied	295	62.37
Average	49	10.36
Dissatisfied	9	1.9
When you're having a hard time, you think your social support is		
Very good	131	27.7
Good	238	50.32
Average	89	18.82
Not so good	15	3.17
Are you under a lot of financial pressure?		
Very much	65	13.74
More	146	30.87
Average	202	42.71
Less	45	9.51
None	15	3.17
Annual salary		
<CNY 100,000	69	14.59
CNY100,000–150,000	173	36.58
CNY160,000–200,000	166	35.1
CNY210,000–250,000	52	10.99
>CNY 250,000	13	2.75
**Religion**		
Do you have any religious beliefs?		
Yes	73	15.43
No	400	84.57

### 3.2 Status quo of clinical nurses' mental workload, adversity quotient, and job engagement

The total score of mental workloads is 78.24 ± 11.65, the scores of the two dimensions are load feeling, and self‐evaluation in order. The total score of AQ is 128.26 ± 15.84, the dimensions are reach, ownership, control, and endurance, and the total score of job engagement is 42.32 ± 7.79. The dimensions are ranked as vigour, dedication, and absorption in one's work. Table 2 describes the scores of mental workloads, AQ, and job engagement.

**TABLE 2:** The Scores of mental workloads, adversity quotient and job engagement of the nurses.

Questionnaires	Dimensions	Total score		Average score	
		Mean	*SD*	Mean	*SD*
Mental workload	Load feelings	52.67	9.31	13.17	2.33
	Self‐assessment	25.58	4.18	12.79	2.09
	Total	78.24	11.65	13.04	1.94
Adversity quotient	Control	31.94	6.03	6.39	1.21
	Ownership	32.07	4.17	6.41	0.83
	Reach	32.29	4.59	6.46	0.92
	Endurance	31.96	4.33	6.39	0.87
	Total	128.26	15.84	6.41	0.79
Job engagement	Vigour	14.77	2.74	4.92	0.91
	Dedication	14.33	2.92	4.78	0.97
	Absorption in one's work	13.34	3.01	4.45	1.00
	Total	42.32	7.79	7.05	1.30

### 3.3 Subgroup comparison of sociodemographic factors in nurses' job engagement

There are significant differences between different subgroups, such as labor‐management relationship with current organization, department of work, satisfaction with marriage, social support, being under a lot of financial pressure, significant stress in the last 6 months, attitude towards a career in Nursing, attitude towards the current career position, the organization provides opportunities for further study to get a degree or diploma in their spare time, religion, See Table 3 for details.

**TABLE 3:** Demographic characteristics of the subjects and subgroup differences with job engagement.

Characteristics	*N*	(mean ± SD)	t or F	p
**General demographic characteristics**				
Gender				
Male	48	41.33 ± 9.24	−0.92	0.356
Female	425	42.43 ± 7.62		
Age				
<25 years	84	41.19 ± 8.60	2.35	0.070
25 years to 35 years	244	42.87 ± 7.86		
36 years and 40 years	89	43.04 ± 6.61		
>40 years	56	40.45 ± 7.66		
Are you an only child?				
Yes	178	42.53 ± 8.23	0.46	0.640
No	295	42.19 ± 7.53		
**Current status of work and study**				
Labor‐management relationship with the current organization				
Have an establishment	304	44.29 ± 7.67	8.14	<0.001
No establishment	169	38.78 ± 6.70		
Length of service in nursing				
<2 years	77	41.74 ± 9.57	0.45	0.770
2–5 years	114	42.54 ± 8.29		
6–10 years	120	42.99 ± 7.57		
11–20 years	112	42.03 ± 6.63		
>20 years	50	41.76 ± 6.61		
Department of Work				
Internal medicine	143	42.81 ± 8.26	4.81	<0.001
Surgery	126	44.83 ± 7.63		
Outpatient clinic	19	39.42 ± 6.43		
Emergency	11	39.27 ± 11.06		
ICU	43	40.37 ± 7.34		
Paediatrics	17	38.88 ± 8.58		
Others	114	40.95 ± 6.40		
Highest level of education you currently have earned				
Technical secondary school degree	11	40.82 ± 3.92	0.86	0.460
Associate degree	20	39.95 ± 6.13		
Bachelor degree	415	42.5 ± 7.76		
Master degree or above	27	41.85 ± 10.21		
Current nursing technical title				
The nurse	75	41.07 ± 8.91	0.89	0.450
Nurse practitioner	176	42.39 ± 8.36		
Nurse‐in‐charge	205	42.76 ± 6.82		
Associate chief nurse and above	17	41.82 ± 7.57		
Have you recently experienced significant stress? (within 6 months)				
Yes	98	39.83 ± 7.55	0.076	<0.001
No	375	42.97 ± 7.74		
Do you have opportunities for study and further education?				
A lot	32	51.63 ± 1.33	24.37	<0.001
More	156	43.78 ± 7.07		
Average	187	41.27 ± 6.99		
Less	82	40 ± 7.41		
None	16	33.69 ± 6.77		
Do you study to get a degree or diploma in your spare time				
Yes	227	44.19 ± 7.80	5.13	<0.001
No	246	40.6 ± 7.40		
**Attitude towards profession and position**				
Your Attitude towards a Career in Nursing				
Like it very much	34	54.12 ± 8.25	56.8	<0.001
Like it more	192	44.69 ± 6.12		
Average	219	39.4 ± 6.31		
Like it less	25	34.72 ± 6.82		
Dislike	3	33.33 ± 7.51		
Attitude towards the current career position				
Very satisfied	34	51.38 ± 6.03	53.35	<0.001
More satisfied	216	44.9 ± 6.90		
Average	195	39.22 ± 6.25		
Less satisfied	13	37.38 ± 6.25		
Dissatisfied	15	29.27 ± 4.04		
Do you think it's difficult to get promoted?				
Difficult	278	41.81 ± 7.90	1.91	0.150
Average	183	43.18 ± 7.58		
Not difficult	12	40.92 ± 7.83		
Do you think Scientific research is stressful?				
Difficult	381	42.31 ± 7.84	0.45	0.640
Average	83	42.59 ± 7.79		
Not difficult	9	40 ± 5.81		
**Social and economic situations**				
Current marital status				
Unmarried	164	42.06 ± 8.79	1.13	0.340
Married	283	42.71 ± 7.24		
Divorced	9	41.22 ± 7.69		
Widowed	6	38.67 ± 7.50		
Remarried	11	38.91 ± 5.34		
Satisfaction with marriage				
Very satisfied	120	46.17 ± 8.89	14.93	<0.001
Be satisfied	295	41.14 ± 7.26		
Average	49	40.9 ± 4.65		
Dissatisfied	9	37.44 ± 5.15		
When you're having a hard time, you think your social support is				
Very good	131	46.59 ± 8.58	27.37	<0.001
Good	238	41.42 ± 6.40		
Average	89	39.96 ± 7.24		
Not so good	15	33.4 ± 6.10		
Are you under a lot of financial pressure?				
Very much	65	45.52 ± 10.92	3.69	0.010
More	146	41.58 ± 8.17		
Average	202	41.67 ± 6.37		
Less	45	43.22 ± 6.48		
None	15	41.73 ± 5.87		
Annual salary				
<CNY 100,000	69	40.23 ± 8.74	1.91	0.110
CNY100,000–150,000	173	42.66 ± 8.02		
CNY160,000–200,000	166	43.1 ± 6.54		
CNY210,000–250,000	52	41.75 ± 9.06		
>CNY 250,000	13	41.08 ± 7.84		
**Religion**				
Do you have any religious beliefs?				
Yes	73	44.42 ± 8.57	2.321	0.022
No	400	41.94 ± 7.59		

### 3.4 Influencing factors of nurses' job engagement

Pearson correlation was used to analyse the relationship between research variables, as shown in Table 4. The total score and dimensions of job engagement were significantly positively correlated with the total score and dimensions of the AQ (*r* = 0.285 ~ 0.618, *p*<0.001), and the total score of mental workload and all dimensions were significantly negatively correlated the total score and dimensions of job engagement (*r* = −0.499 ~ −0.319, *p*<0.001). The total score and dimensions of mental workload were negatively correlated with the total score and dimensions of the AQ (*r* = −0.330 ~ − 0.113, *p*< 0.05).

**TABLE 4:** Correlation analysis of mental workload, AQ and job engagement.

Variables	1	2	3	4	5	6	7	8	9	10	11	12
1	1											
2	0.842**	1										
3	0.838**	0.740**	1									
4	0.815**	0.643**	0.619**	1								
5	−0.499**	−0.475**	−0.449**	−0.403**	1							
6	−0.417**	−0.414**	−0.416**	−0.319**	0.945**	1						
7	−0.463**	−0.400**	−0.323**	−0.412**	0.683**	0.405**	1					
8	0.613**	0.552**	0.539**	0.526**	−0.291**	−0.243**	−0.269**	1				
9	0.576**	0.501**	0.463**	0.514**	−0.314**	−0.280**	−0.252**	0.877**	1			
10	0.342**	0.285**	0.348**	0.288**	−0.180**	−0.175**	−0.113*	0.739**	0.503**	1		
11	0.464**	0.461**	0.404**	0.388**	−0.116*	−0.061	−0.188**	0.842**	0.625**	0.540**	1	
12	0.618**	0.559**	0.562**	0.519**	−0.330**	−0.267**	−0.325**	0.833**	0.669**	0.465**	0.629**	1

Note: 1 ~ 12 represent the job engagement, vigour, dedication, absorption in one^'^s work, mental workload, load feelings, self‐assessment, AQ, control, ownership, reach, and endurance, respectively.

** Represent *p* < 0.01.

* Represent *p* < 0.05.

Linear regression analysis was used to evaluate the effects of demographic characteristics, mental workload, and AQ on job engagement. With job engagement as the dependent variable, and demographic characteristic variable, mental workload, and AQ dimensions as the independent variables, the settings, and assignments of dummy variables with demographic characteristics are all shown (see File S2). There is no obvious multicollinearity in the model. The tolerance of the model is 0.357 ~ 0.816 (>0.10), and the Variance inflation factor (VIF) is 1.226 ~ 2.801 (< 10). The results showed that the labor‐management relationship with the current organization, department, study to get a degree or diploma in spare time, attitude towards a career in Nursing, attitude towards the current career position, satisfaction with marriage, social support, load feelings, self‐assessment, control, and endurance had predictive effects on nurses' job engagement. (*F* = 31.291, *p* < 0.01; *R* ^2^ = 0.733, Adjust‐*R* ^2^ = 0.709). The results of linear regression analysis of job engagement are shown in Table 5.

**TABLE 5:** The Liner Regression Analysis results for job engagement.

Variables	B	Beta	*t*	*p*	95% confidence interval	
					Lower bound	Upper bound
(Constant)	42.951		13.163	0.000	36.538	49.364
Current status of work and study						
Labor‐management relationship with current organization(No establishment)	−1.346	−0.083	−2.814	0.005	−2.286	−0.406
Department of work(Internal medicine)	−1.629	−0.096	−2.887	0.004	−2.739	−0.520
Department of work(Outpatient clinic)	−2.452	−0.062	−2.196	0.029	−4.646	−0.257
Department of work(Emergency)	−2.794	−0.054	−1.711	0.088	−6.004	0.415
Department of work(ICU)	−2.600	−0.096	−3.233	0.001	−4.180	−1.019
Department of work (Paediatrics)	−3.006	−0.072	−2.445	0.015	−5.423	−0.589
Department of work (Others)	−2.503	−0.138	−4.167	0.000	−3.684	−1.322
Have you recently experienced significant stress? (within 6 months) (Yes)	−0.032	−0.002	−0.054	0.957	−1.189	1.126
Do you have opportunities for study and further education? (A lot)	0.404	0.013	0.401	0.689	−1.575	2.382
Do you have opportunities for study and further education? (More)	−0.573	−0.035	−1.051	0.294	−1.644	0.498
Do you have opportunities for study and further education? (Less)	−0.878	−0.043	−1.426	0.155	−2.087	0.332
Do you have opportunities for study and further education? (None)	−2.423	−0.056	−1.799	0.073	−5.071	0.224
Do you study to get a degree or diploma in your spare time? (No)	−2.341	−0.150	−5.350	0.000	−3.201	−1.481
Attitude towards profession and position						
Your Attitude towards a Career in Nursing (Like it very much)	4.847	0.161	4.456	0.000	2.709	6.985
Your Attitude towards a Career in Nursing (Like it more)	1.750	0.110	3.282	0.001	0.702	2.798
Your Attitude towards a Career in Nursing (Like it less)	−0.935	−0.027	−0.742	0.459	−3.412	1.543
Your Attitude towards a Career in Nursing (Dislike)	0.133	0.001	0.041	0.967	−6.221	6.486
Attitude towards the current career position (Very satisfied)	−0.072	−0.002	−0.080	0.936	−1.851	1.706
Attitude towards the current career position (Average)	−2.922	−0.185	−5.536	0.000	−3.959	−1.884
Attitude towards the current career position (Less satisfied)	−1.187	−0.025	−0.753	0.452	−4.286	1.912
Attitude towards the current career position (Dissatisfied)	−7.158	−0.161	−4.353	0.000	−10.391	−3.926
Social and economic situations						
Satisfaction with marriage (Very satisfied)	1.066	0.060	1.786	0.075	−0.107	2.239
Satisfaction with marriage (Average)	1.310	0.051	1.803	0.072	−0.118	2.738
Satisfaction with marriage (Dissatisfied)	−1.782	−0.031	−0.963	0.336	−5.418	1.855
Social support (Good)	−1.730	−0.111	−2.939	0.003	−2.887	−0.573
Social support (Average)	−0.802	−0.040	−1.075	0.283	−2.268	0.664
Social support (Not so good)	0.023	0.001	0.013	0.990	−3.604	3.651
Are you under a lot of financial pressure? (Very much)	1.510	0.067	1.940	0.053	−0.020	3.040
Are you under a lot of financial pressure? (Average)	−0.017	−0.001	−0.034	0.973	−1.009	0.974
Are you under a lot of financial pressure? (Less)	−0.612	−0.023	−0.764	0.445	−2.188	0.963
Are you under a lot of financial pressure? (None)	1.602	0.036	1.175	0.241	−1.077	4.281
Religion						
Do you have any religious beliefs? (No)	−0.349	−0.016	−0.589	0.556	−1.513	0.815
Mental workload						
Load feelings	−0.093	−0.111	−3.500	0.001	−0.145	−0.041
Self‐assessment	−0.398	−0.214	−6.868	0.000	−0.512	−0.284
Adversity quotient						
Control	0.166	0.129	3.266	0.001	0.066	0.267
Ownership	0.029	0.016	0.475	0.635	−0.092	0.150
Reach	0.071	0.042	1.073	0.284	−0.059	0.200
Endurance	0.339	0.188	4.661	0.000	0.196	0.482
R2 (adjust R2)	0.733 (0.709)					
F (*P*)	31.291 (<0.001)					

## 4 DISCUSSION

During the COVID‐19 epidemic, clinical nurses are under more significant pressure and responsibility than usual, and are prone to negative emotions, such as anxiety and depression. However, nurses in Grade‐A tertiary hospitals are not only engaged in clinical nursing, but also shoulder teaching, scientific research, and other tasks, and their workload is greater than ever. Therefore, it is essential to understand the influencing factors of nurses' job engagement in Grade‐A tertiary hospitals for efficient intervention to improve it.

The mental workload score of nurses in this study was higher than the median value (5 × 10 = 50), which was also higher than the result of Liu and Dai's study (Dai et al., 2023; Liu et al., 2022) The result is consistent with the working pressure on nurses was at a moderately high level during the epidemic (Zheng et al., 2020). Considering that it was related to the severity of the epidemic caused by the time difference in the study, and population density caused by regional differences, it was worth noting that nurses generally felt a heavy workload, which exceeded the evaluation of individual behaviour. This survey found that clinical nurses' AQ score was medium, points are scored in order of arrival reach, ownership, control, and endurance, similar to Wang's study (Wang, Liu, et al., 2021) on nursing students in Macau, but the AQ of clinical nurses was higher than that of nursing students. The job engagement scores of nurses in this study were higher than the median (3 × 9 = 27), higher than the results of a study on dental nurses (Wang, Gao, & Xun, 2021), and higher than the scores of clinical nurse experts and general nurses in Shandong Province when the epidemic occurred (Zhang, Chen, et al., 2023), it might be due to regional differences in methodological or sampling differences. The dimensional ranking was consistent with (Wang et al., 2017) study on ICU nurses. The consideration of the vigour dimension is highest is related to the younger age of this sample, but the absorption in one's work dimension is low.

This study found no difference in gender, age, marital status, length of service in nursing, current Nursing technical title, which was similar to the results of Hu's research (Hu et al., 2022). First of all, it was found that nurses with no establishment were less engaged in work. Considering that these nurses were worse than nurses having an establishment in terms of welfare benefits, different pay for the same work, and occupational instability might trigger the intention of nurses to change their professional places, the job engagement of these nurses was reduced. Secondly, this study found that the job engagement of surgical nurses was higher than that of nurses in other departments, which was different from Wang's study (Wang, Liu, et al., 2021) that there was no difference between departments. This may be related to the short hospital stay of surgical patients, high admission and discharge rate, and relatively high salaries of nurses (In Chinese hospitals, the fast turnover of patients means that a large amount of examination fees are charged in the first 3 days, thus maximizing the benefits of the department, and the hospital). The buffering hypothesis showed that nurses face high job requirements in clinical work. However, much more clinical nursing knowledge, and rich labor remuneration can be used as beneficial psychological capital, and job resources to effectively buffer the loss of employees with high job requirements, and increase job engagement (Zhou et al., 2022). Internal medicine, emergency, and other departments have a negative effect on predicting job engagement. Considering that the internal medicine wards are full of chronic patients with long disease courses, low turnover rates, complex work content, and mismatched income, resulting in low job engagement. The emergency department has put forward higher requirements and challenges for nurses' knowledge, skills, and emergency response‐ability, and nurses who are in a tight state for a long time are also prone to burnout. The rapid progress, and change of paediatric diseases, coupled with the easy occurrence of nurse–patient conflicts in paediatrics, increase paediatric nurses' job burnout, and affect their job engagement. However, nurses in other departments, such as the supply room, and endoscopy room, work relatively easily, with little application of clinical nursing knowledge, and skills, and limited job engagement.

Thirdly, nurses who studied to get a degree or diploma in their spare time have higher levels of job engagement. The nurses can further enrich their professional knowledge, and improve their professional skills through further study, thus forming positive feedback, and improving their work efficiency, job satisfaction, and get more job engagement in work. A study found that job resources, such as employee empowerment, management support, feedback, and development opportunities, and personal resources, such as self‐efficacy, and optimism, are predictive factors of job engagement (Van Heerden et al., 2022). According to the JD‐R model, learning, and further study, these positive job factors that promote personal growth, learning, and development are all job resources that can encourage job engagement.

Fourthly, the results of this study showed that nurses' satisfaction with their nursing careers, and their current position was closely related to their job engagement. Regression analysis showed similar results. This is consistent with the research results of Zou (Zou et al., 2019). A higher sense of professional identity can form positive emotions, and stimulate the clinical work of nurses, thus improving the level of job engagement in clinical nursing work. Occupational identity can promote job satisfaction through the single mediating effect, and chain mediating effect of psychological empowerment, and job engagement (Sun et al., 2022).

Fifthly, nurses with higher satisfaction with marriage and higher social support had higher levels of job engagement. Support from family and society is a job resource that promotes job engagement. Family support can provide individuals with emotional dependence, and relaxation experience, help individuals resist pressure, improve happiness, and maintain physical, and mental health, then improve the level of job engagement correspondingly (Ma et al., 2020). Studies have shown that social support, performance feedback, skill diversity, autonomy, and learning opportunities from managers and colleagues are positively correlated with job engagement (Van Heerden et al., 2022). Studies (Liang et al., 2022) have shown that the higher the sense of organizational support nurses feel, the higher level of job engagement.

Nurses with religions are more engaged in their work, considering that the altruistic attribute of religion has a certain promoting effect on the service consciousness of nursing work. Some literature showed that job engagement is closely related to financial return, job performance, turnover intention, and employee creativity (Xie et al., 2023; Li et al., 2022;). Nurses with tremendous financial pressure had a higher level of job engagement. Considering that Hangzhou is a city with high housing prices and consumption, many nurses are under financial stress, such as mortgage repayment, and are more afraid of unemployment, or losing stable salary support, so they are more cautious about quitting. Nurses who have recently experienced significant stress also have low job engagement. Considering the impact of stress on the body and mind, nurses' job engagement is bound to be affected. Although these three factors have an impact on job engagement alone, they cannot be included in the regression equation when combined with other socio‐demographic factors. Therefore, it is very necessary to advocate equal pay for equal work, and break the differences brought by the personnel establishment. Except for the surgery department, all other clinical departments negatively predict the job engagement of nurses, indicating that clinical nurses have experienced high pressure, and job burnout. Therefore, providing more opportunities for nurses to further their studies for the development of their nursing career is a vital thing. At the same time, it is crucial to strengthen nurses' professional identity and love, improve their work remuneration, and welfare benefits, reduce the economic pressure on nurses in Grade‐A tertiary hospitals according to the different departments, and provide more spiritual and material support.

Workload includes objective and subjective workload. Subjective workload refers to the workload perceived by medical staff, and is often measured by their personal feelings (Han et al., 2022). The mental workload was often linked to human performance, and mental health (Radüntz, 2020). The mental workload can indirectly lead to fatigue through stress, and emotional disorders, thus highly predicting the level of job engagement (Pace & Sciotto, 2021). The study found that mental workload was negatively correlated with job engagement in this study. The result was consistent with Wang's study (Wang et al., 2020), which found that job engagement of clinical nurses increased by 0.331 units for every 1‐unit reduction in department‐level workload after adjusting nurses' population, and occupation factors. Both correlation and regression analysis showed that self‐assessment of their mental workload had a greater impact on job engagement, that is, even in the face of greater mental, physical, and work time pressure, the better self‐performance of nurses in work, the lower the frustration, and the higher the job engagement. This result indicated that nurses' subjective workloads, such as relative working hours, nurse–patient conflict, and work difficulty, have a more significant impact on job engagement than objective loads (nurse–patient ratio, labor intensity, and working hours). The more the candidate's self‐performance failure, frustration feeling more vigorous, as a negative factor in the work consumption of individual energy, and increased psychological cost related to the job requirements, is bound to affect their job engagement. Nursing managers are advised to guide the nurses to reinforce the correct perception and evaluation of pressure, and cope with work pressure actively.

In this study, AQ is positively correlated with job engagement, consistent with Safi'i's research results (Safi'i et al., 2021). The regression analysis also confirmed that the control, and endurance had a great influence on job engagement. People with high AQ have high mental resilience in the face of adversity (Khampirat, 2020), they will show a positive, and optimistic attitude, and dare to accept challenges (Wu et al., 2022). Therefore, they can persevere, and become more courageous after setbacks. Individuals with high AQ would think that adversity would not exist for a long time, and they believe that things are getting better (Schafer et al., 2011), and they can effectively face obstacles, and take advantage of opportunities (Safi'i et al., 2021). So they have good adaptability, thus reducing the consumption of internal job resources, and promoting performance (job engagement) (Safi'i et al., 2021). Therefore, it is very important to increase the control of nurses, and reduce the reach, and endurance of adversity. If the reality of adversity cannot be changed, staff deployment, or staff rotation can be properly carried out to buffer the high workload (Nasirizad Moghadam et al., 2021).

In summary, improving the AQ of clinical nurses is the key to promoting the development of nursing work. Therefore, reasonable evaluation of mental workload, strengthening the ability of control, enhancing responsibility, and cultivating the positive understanding of the reach, and endurance of adversity can effectively promote the mental health of clinical nurses, improve work enthusiasm, and job engagement, to ensure the quality of nursing. Firstly, it should reduce the subjective mental workload of nurses. We can take steps to reduce time pressure, and emotional workload, and avoid information overload (Arnold et al., 2023); More sustainable support can be provided to reduce the mental workload either (Akrour et al., 2022). Yang adopted the Balint group mode to reduce the mental workload caused by the professional pressure of nurses in the neonatal intensive care unit, and improve the satisfaction of nurses with their work (Yang et al., 2020). Workload management programs should be an effective way to reduce mental workload, and enhance nurses' job engagement (Mohamadi et al., 2019). Establishing proper mentoring programs, and employing more experienced nursing professionals may be another method to reduce it (Zahednezhad et al., 2021); Emotional intelligence educational programs could decrease the level of mental workload(Mohamadi et al., 2019); Secondly, it should improve the training of nurses' anti‐quotient ability, especially the control ability. In previous studies, there were mainly three ways to improve AQ. First, courses related to adversity quotient were carried out, such as embedded adversity training education could improve the AQ level of nursing undergraduates (Liu et al., 2021), and correct attribution education might be useful to improve AQ (Wang & Mei, 2016). AQ‐related courses are also developed to master the psychological characteristics of different nurses, and take personalized measures to improve nurses' anti‐frustration ability (Zhao & Li, 2019). Secondly, when encountering adversity, nurses should be channelled negative psychological emotions timely, and conduct regular psychological interventions to relieve mental pressure, such as the focused solution mode can eliminate the bad emotions of nurses well, and give nurses psychological counselling in time, to strengthen their self‐confidence (Song et al., 2020), and improve their coping ability; Reducing the level of fatigue is also a good way to reduce the negative emotions of nurses (Sun et al., 2023); Finally, it should improve nurses' professional pride, enhance their professional identity, realize their self‐worth. For example, Gender Equity in Nursing Education Programs is helpful in improving nurses' professional pride (Shim & Park, 2023), and the goal incentive method can enhance nurses' professional pride, and professional performance effectively (Shim & Park, 2023).

### 4.1 Limitations

First, it is essential to note that this study only included nurses from Grade‐A tertiary hospitals in Hangzhou as subjects. It lacks nurses sampling from primary hospitals, secondary hospitals, and community hospitals, and lacks sample sources from the cities of under‐developed provinces and regions in China. This study cannot represent Chinese regions with different economic and medical conditions, which is relatively limited. Second, a self‐report questionnaire was adopted, lacking objective indicators. Caution should be exercised in extrapolating findings on the effects and relationships between sociodemographic factors, mental workload, and AQ on job engagement in other regions. Finally, only some demographic characteristics, mental workload (job requirements), and AQ (job resources) were selected to explore the influencing factors of nurses' job engagement in China's Grade‐A tertiary hospitals. Fourth, too many sociodemographic independent variables are included, making interactions difficult to analyse. As for the JD‐R model, the research content has certain limitations. The research team will further explore other influencing factors of job engagement of hospital nurses, and explore the practical effects of some interventions actively.

### 4.2 Conclusions

In this study, it was found that in the context of emergencies such as the outbreak of the epidemic, the mental workload of nurses in tertiary, A‐grade hospitals increased significantly, their AQ was at a medium level, and their job engagement also increased to a certain extent. The results showed that: labor‐management relationship with current organization, department, study to get a degree or diploma in spare time, attitude towards a career in Nursing, attitude towards the current career position, satisfaction with marriage, social support, load feelings, self‐assessment, control, and endurance could predict 70.9% of job engagement. The results of this study have positive significance for guiding nurses with different socio‐demographic characteristics to improve job engagement. At the same time, it is also necessary to guide nurses in Grade‐A tertiary hospitals to view their job demands(workload) from an objective and reasonable perspective, reduce their mental workload, adopt more effective measures to improve their job resource (AQ), and then provide nurses with material, and spiritual support, and opportunities for learning according to the influencing factors of social‐demographic data. More attention should be paid to the physical, and mental health of nursing staff. In the future, the interventions and effects of nursing managers in these areas should be monitored and evaluated continually.

## AUTHOR CONTRIBUTIONS

All authors agreed on the final version and meet at least one of the following criteria. Make a significant contribution to concept and design, data acquisition or data analysis and interpretation; Draft articles or revise important intellectual content critically.

## FUNDING INFORMATION

This research received funding from scientific research project of education department of Zhejiang province in 2020(Y202045141), national entrepreneurship training program for College Students of Zhejiang Province in 2020 (202011842026).

## CONFLICT OF INTEREST STATEMENT

The authors declare that they have no known competing financial interests or personal relationships that could have appeared to influence the work reported in this paper.

## ETHICS STATEMENT

The protocol for this study was approved by the Ethics Review Committee of the Institutional Review Board of Zhejiang Shu Ren University (NO:20221022). The research process was conducted under the Declaration of Helsinki and its later amendments.

## Data Availability

The raw data set analysed in the current study is available from the corresponding author (Lili Yang) on reasonable request.
